# Efficient hybrid algorithm for nonnegative matrix factorization based on modified nonmonotone linear search

**DOI:** 10.1371/journal.pone.0344857

**Published:** 2026-07-30

**Authors:** Jing Wu, Wenbo Li, Lijun Su, Huiru Wang, Yike Li

**Affiliations:** 1 School of Mathematics, Xi’an University of Technology, Xi’an, China; 2 School of Computer Science, Xijing University, Xi’an, China; 3 Department of Thyroid and Breast Surgery, Xijing Hospital of Air Force Military Medical University, Xi’an, China; Northwestern Polytechnical University, CHINA

## Abstract

In this paper, we present a modified nonmonotone line search algorithm that employs a variable parameter to control the degree of nonmonotonicity. This modification enhances both the probability of identifying the global minimum and the rate of convergence. Within the framework of alternating nonnegative least squares (ANLS), we propose a hybrid algorithm that employs either the modified nonmonotone projected Barzilai–Borwein method and the block coordinate descent method to address the subproblems in each iteration. To further accelerate convergence, we integrate a technique that allows for a larger step size. Under mild assumptions, we establish the global convergence of the algorithm. Numerical experiments conducted on both synthetic and real datasets demonstrate that the proposed algorithm is efficient for nonnegative matrix factorization (NMF) and outperforms other state-of-the-art methods.

## 1 Introduction

Nonnegative Matrix Factorization (NMF) [1–5] is a prevalent and efficacious technique for the analysis of high-dimensional datasets. It facilitates the automatic extraction of sparse and significant features from a set of nonnegative, redundant data vectors. The standard NMF problem is formulated as follows: given an m×n data matrix *V* with $V_{ij}\geq 0$ and a pre-specified positive integer r<min{m,n}, NMF seeks two nonnegative matrices $W\in {\mathbb{R}}_{+}^{m\times r}$ and $H\in {\mathbb{R}}_{+}^{r\times n}$ such that


$$\begin{array}{c} V=WH+E, \end{array}$$ (1)

where $E\in R^{m\times n}$ denotes the approximation error. A common formulation for solving NMF (1) is


$$\begin{array}{c} \begin{array}{c} \min_{W,H}f(W,H)\equiv \frac{1}{2}\|V-WH{\|}_{F}^{2}\\ \text{subject to}\;W\geq 0,H\geq 0, \end{array} \end{array}$$ (2)

where $\|\cdot {\|}_{F}$ is the Frobenius norm. This model is particularly suitable for the problem (1) when the approximation error follows Gaussian noise, which is a common assumption in many practical applications [6].

The NMF problem (2), initially introduced by Paatero and Tapper [5], has attracted significant attention in recent years owing to its broad applicability in areas such as image processing [7], text mining [8], machine learning [9], and subsystem identification [10].

We now briefly review several well-established approaches for solving the NMF problem (2). Lee and Seung developed a multiplicative update (MU) algorithm [3] and showed its equivalence to a gradient descent method. However, a limitation of the MU algorithm is that it may not converge to a stationary point due to the possibility of division by zero in the update rules [11]. To address this, Pauca et al. [8] introduced a small positive constant into the update rules, leading to improved numerical performance.

The Alternating Nonnegative Least Squares (ANLS) framework [5] provides another major approach, with proven convergence to a stationary point of (11). Within this framework, several methods have been developed, such as the projected gradient method by Lin [12] and a quasi-Newton method by Kim et al. [2]. Other contributions include the alternating projected Barzilai–Borwein methods from Han et al. [13] and a projected Newton method by Gong and Zhang [1].

Despite the variety of methods available for solving NMF problem (2), many suffer from inefficiencies due to challenges in selecting line search step sizes or inverting Hessian matrices, especially for large-scale applications. Recently, several fast algorithms based on proximal point modifications have been proposed, such as NeNMF [14], quadratic regularization projected Barzilai–Borwein [15], and monotone projected Barzilai–Borwein (MPBB) [16]. Guan et al. [14] introduced NeNMF, which leverages Nesterov’s optimal gradient method (OGM) [17] without line search. However, Huang et al. [15] noted that OGM may require a large number of iterations to achieve a given tolerance, limiting the efficiency of NeNMF. The QRPBB method [15] utilizes the Lipschitz constant of the gradient to enhance performance and has been shown to outperform other solvers such as PG [12], APBB2 [13], and NeNMF [14]. Nevertheless, QRPBB involves a computationally expensive nonmonotone line search. To address this, the MPBB method [16] was proposed, which determines the step size without line search. However, our analysis indicates that MPBB still requires many iterations to meet a given tolerance, which can be prohibitive for large-scale problems. These limitations motivate the development of a more efficient algorithm for NMF.

In this paper, we introduce a modified nonmonotone technique based on a variable parameter that controls the degree of nonmonotonicity in the line search rule. We theoretically prove that the modified objective function forms a decreasing sequence, which aids in establishing global convergence under mild conditions. By incorporating this modified nonmonotone line search, we propose an efficient hybrid algorithm that employs either the projected gradient method or the block coordinate descent method to solve subproblems within the ANLS framework. The Barzilai–Borwein step size and a larger step size technique are utilized to accelerate convergence. We establish the global convergence of the proposed method. Numerical experiments on synthetic, image, and text datasets demonstrate the efficiency of our algorithm.

The remainder of this paper is organized as follows. Section 2 introduces the fast hybrid algorithm for NMF and presents its global convergence analysis. Section 3 provides experimental comparisons with other NMF algorithms. Finally, Section 4 concludes the paper.

## 2 A hybrid method for NMF and its global convergence

In this section, we first propose a modified nonmonotone line search technique, and then develop a fast hybrid method for NMF by incorporating this technique along with a strategy for enabling larger step sizes. We also establish the global convergence of the proposed algorithm.

Note that since the role of *W* and *H* is perfectly symmetric for the problem (2), therefore, we focus only on the update of the matrix *W*. Suppose that the matrices $V\in R_{+}^{m\times n}$ and $H_{k}\in R_{+}^{r\times n}$ are fixed, we only need to solve the following problems:


$$\begin{array}{c} \min_{W\geq 0}f(W,H^{k})=\frac{1}{2}\|V-WH^{k}{\|}_{F}^{2}. \end{array}$$ (3)

To simplify the notation, $f(W,H^{k})$ will be denoted simply by *f*(*W*) in the following part of this paper.

It is easy to know that the objective function *f*(*W*) is convex [18–22] and the gradient of it is Lipschitz continuous by Lemma 1 and 2 in [14]. These properties are very important in our proof, which will be presented in following result.

**Lemma 1** [14] The following two statements are valid.

(i) The objective function f(W) of (3) is convex.
(ii) The gradient

$$\begin{array}{c} \nabla f(W)=(WH^{k}-V)(H^{k}{)}^{T} \end{array}$$

is Lipschitz continuous with constant $L=\|H^{k}(H^{k}{)}^{T}{\|}_{2}$.

Since cost function (3) is not strongly convex objective function, and thus one can only expect that the algorithms find a stationary point instead of the global or even local minimizer. To overcome this drawback, similar to [23], in this paper, we consider a proximal point modification version of cost function (3) by linearizing *f*(*W*) which is the form of


$$\begin{array}{c} \min_{W\geq 0}\varphi (\tilde{W},W):=\langle \nabla f(\tilde{W}),W-\tilde{W}\rangle +\frac{L}{2}\|W-\tilde{W}{\|}_{F}^{2}. \end{array}$$ (4)

Obviously, according to (ii) of the Lemma 1, we can deduce that $\varphi (\tilde{W},W)$ is strictly convex in *W* for any fixed $\tilde{W}$. At each iteration, we first calculate a point $Z_{t}$ by solving the following strongly convex quadratic minimization problem


$$\begin{array}{c} \min_{W\geq 0}\varphi (W_{t},W). \end{array}$$ (5)

Due to the strongly convex property of the objective function (4), the problem has a unique closed-form solution


$$\begin{array}{c} Z_{t}=P[W_{t}-\frac{1}{L_{W}}\nabla_{W}f(W_{t},H^{k})], \end{array}$$ (6)

where $L_{W}=\|W^{k}(W^{k}{)}^{T}{\|}_{2}$ and the operator *P*[*X*] projects all the negative entries of *X* to zero.

In next section, we will put forward an efficient hybrid strategy to update $W_{t+1}$ by using $Z_{t}$.

### 2.1 Main algorithm

In [15], the authors developed a nonmonotone proximal quadratic regularization projected alternating Barzilai-Borwein method for NMF, incorporating a nonmonotone technique and the Barzilai-Borwein step-size [24].

This nonmonotone term, adapted from [25], defines


$$\begin{array}{c} C_{t}=\max_{0\leq j\leq \text{min}\{t,M-1\}}f(W_{t-j}), \end{array}$$ (7)

where *M* is a predefined constant.

Because the maximum function may discard favorable function values from any iteration, the method’s numerical performance can depend significantly on the choice of *M*.

To overcome these limitations, Zhang and Hager proposed an alternative nonmonotone technique in [26], where a weighted average of previous function values replaces the maximum function value. This approach relaxes the Armijo-type line search condition as follows:


$$\begin{array}{c} C_{t}=\left\{ \begin{array}{cc} f(W_{t}), & \text{if}\;t=0,\\ (\eta_{t-1}Q_{t-1}C_{t-1}+f(W_{t}))/Q_{t}, & \text{if}\;t\geq 1, \end{array}\right. \end{array}$$

with


$$\begin{array}{c} Q_{t}=\left\{ \begin{array}{cc} 1, & \text{if}\;t=0,\\ \eta_{t-1}Q_{t-1}+1, & \text{if}\;t\geq 1, \end{array}\right. \end{array}$$

where $\eta_{t-1}\in [\eta_{min},\eta_{max}]$ for $\eta_{min}\in [0,1)$ and $\eta_{max}\in [\eta_{min},1]$. Clearly, $C_{t}$ represents a convex combination of the preceding $C_{t-1}$ and $f(W_{t})$, incorporating the complex terms $\eta_{t}$ and $Q_{t}$. This formulation implies that each $C_{t}$ implicitly incorporates information from all previously known function values. In practice, however, updating $\eta_{t}$ and $Q_{t}$ at every iteration becomes computationally burdensome. To address this limitation, the following expression for $S_{t}$ [27] was proposed:


$$\begin{array}{c} S_{t}=\left\{ \begin{array}{cc} f(W_{0}), & \text{if}\;t=0,\\ f(W_{t})+\eta_{t-1}(S_{t-1}-f(W_{t})), & \text{if}\;t\geq 1, \end{array}\right. \end{array}$$ (8)

using either a fixed η∈(0,1) or a variable $\eta_{t}$.

An analysis of $S_{t}$ (8) reveals that it fails to fully utilize the current well function value. Notably, the nonmonotone strategy exhibits superior performance by incorporating the maximum function—this can be attributed to the fact that the maximum function represents one of the most prominent pieces of information derived from recent successful iterations, a factor that should not be overlooked. Consequently, we define


$$\begin{array}{c} \begin{array}{c} S_{l(t)}=\max_{0\leq j\leq m(t)}\{S_{t-j}\}, \end{array} \end{array}$$ (9)

with 0≤m(t)≤min{m(t−1)+1,M}, *m*(0)=0, and


$$\begin{array}{c} S_{t}=\left\{ \begin{array}{cc} f(Z_{0}), & \text{if}\;t=0,\\ f(Z_{t})+\eta_{t-1}(S_{t-1}-f(Z_{t})), & \text{if}\;t\geq 1, \end{array}\right. \end{array}$$ (10)

where $\eta_{t}\in [0,1]$. As with *M* in (7), the selection of $\eta_{t}$ critically influences the degree of nonmonotonicity (see [27]). To enhance the efficiency of the nonmonotone line search, Ahookhosh et al. [28] introduced a varying $\eta_{t}$ determined by a simple formula.

Subsequently, Nosratipour et al. [29] argued that $\eta_{t}$ should relate to a suitable measure of the distance to the optimal solution, defining it as


$$\begin{array}{c} \eta_{t}=1-e^{-\|\nabla f(Z_{t})\|}. \end{array}$$ (11)

However, if the iterative sequence $\left\{Z_{t}\right\}$ becomes trapped in a narrow curved valley, $\nabla f(Z_{t})$ may approach zero, forcing $\eta_{t}$ to zero and reducing the nonmonotone line search to the standard Armijo method, which is inefficient due to very short or zigzagging steps. To address this limitation, we propose the following $\eta_{t}$:


$$\begin{array}{c} \eta_{t}=1-e^{-|f(Z_{t})-f(Z_{t-1})|}. \end{array}$$ (12)

When the function value exhibits a rapid decrease, the absolute difference $\left|f(Z_{t})-f(Z_{t-1})\right|$ increases significantly. Accordingly, this induces an elevation in $\eta_{t}$ and enhances the prominence of the nonmonotone strategy. Conversely, as $f(Z_{t})$ converges toward the optimal solution, $\left|f(Z_{t})-f(Z_{t-1})\right|$ tends to zero. This subsequent reduction in the absolute difference drives $\eta_{t}$ to approach zero, with the nonmonotone strategy gradually losing its efficacy—thereby ultimately transitioning into a monotone strategy.

We then modify the Armijo-type line search by replacing $S_{t}$ [27] with $S_{l(t)}$:


$$\begin{array}{c} f(Z_{t}+\lambda_{t}D_{t})\leq S_{l(t)}+\gamma \beta \lambda_{t}\langle \nabla f(Z_{t}),D_{t}\rangle . \end{array}$$ (13)

Nonmonotone line search methods are known to be efficient for large-scale optimization problems [26–29]. However, a direct application to NMF does not guarantee convergence or the existence of an optimal step size, since the theoretical analysis requires $f(Z_{t})\leq S_{l(t)}$ at each iteration (see Lemma 7 and Theorem 1). A key challenge is therefore how to apply the modified nonmonotone line search technique to (4). Numerical experiments indicate that the modified nonmonotone line search performs well provided $f(Z_{t})\geq S_{t-1}$ and $f(W_{t})\leq S_{t}$ hold. From these conditions and the definition of $S_{l(t)}$, it follows that $f(Z_{t})\leq S_{l(t)}$ (see Lemma 6). It is therefore reasonable to employ the modified nonmonotone line search for solving (4) when $f(Z_{t})\geq S_{t-1}$ and $f(W_{t})\leq S_{t}$; otherwise, we use the block coordinate descent method [30] to solve the NMF.

Based on this discussion, we propose a hybrid strategy for updating $W^{k}$, as detailed in Algorithm 1. The same procedure can be applied to solve for $H^{k}$.

**Algorithm 1. An efficient hybrid method for negative matrix factorization (HMNMF)**

1.  *Initialize* $\alpha_{0}=1$*, M > 0,* $\rho ,\gamma \text{ and }\eta_{t}\in (0,1)$, β∈(1,2), $\alpha_{max}>\alpha_{min}>0$, $\lambda_{max}>0$, $\lambda \leq \lambda_{max}$, $L=\|H^{k}(H^{k}{)}^{T}{\|}_{2}$ *and* $W_{1}=Z_{0}=W_{0}=W^{k}$*. Set t = 1.*

2.  *If* $\|P[W_{t}-\nabla_{W}F(W_{t})]-W_{t}\|=0$*, stop.*

3.  *Compute*

$Z_{t}=P[W_{t}-\frac{1}{L}\nabla_{W}F(W_{t})].$ (14)

4.  *Compute* $S_{t}$ *by (10), and compute* $S_{l(t)}$ *by (9).*

5.  *If* $sgn(f(W_{t})-S_{t})*sgn(f(Z_{t})-S_{t-1})<0$*, set* $W_{t+1}=Z_{t}$ *and go to Step 6. Otherwise, compute*

$D_{t}=P[Z_{t}-\alpha_{t}\nabla f(Z_{t})]-Z_{t}$

*which is the direction with* $\alpha_{t}$ *be the BB stepsize, and let* $m_{t1}$ *and* $m_{t2}$ *are the smallest nonnegative integer m respectively satisfying*

$S_{t}\leq S_{l(t-1)}+(1-\eta_{t-1})\gamma \beta {\tilde{\lambda }}_{t-1}\langle \nabla f(Z_{t-1}),D_{t-1}\rangle ,$ (15)

*with* ${\tilde{\lambda }}_{t-1}=\lambda \rho^{m_{t1}}$ *and*

$f(Z_{t}+\beta \lambda_{t}D_{t})\leq S_{l(t)}+\gamma \beta \lambda_{t}\langle \nabla f(Z_{t}),D_{t}\rangle ,$ (16)

*where* $\lambda_{t}={\tilde{\lambda }}_{t-1}\rho^{m_{t2}}$. *Calculate* $W_{t+1}=Z_{t}+\beta \lambda_{t}D_{t}$.

6.  *Calculate* $S_{t}=W_{t+1}-Z_{t}$ and $Y_{t}=\nabla_{W}F(W_{t+1})-\nabla_{W}F(Z_{t})$. *If* $\langle S_{t},S_{t}\rangle /\langle S_{t},Y_{t}\rangle \leq 0$, *set* $\alpha_{t+1}=\alpha_{max}$; *otherwise, set* $\alpha_{t+1}=\min\{\alpha_{max},\max\{\alpha_{min},\langle S_{t},S_{t}\rangle /\langle S_{t},Y_{t}\rangle \}\}$.

7.  *Set t = t + 1 and go to step 2.*

For ease of reference, we define two index sets:


$$\begin{array}{c} I=\{t:f(W_{t})\leq S_{t}\text{ and }f(Z_{t})\geq S_{t-1}\},\text{ and }J=P\backslash I, \end{array}$$

Here, *P* denotes the index set for the total number of sub-iterations used to solve (4).

**Remark 1** Equation (9) *indicates that the sequence* $\left\{S_{t}\right\}$ *produced by Algorithm 1 can occasionally increase. This behavior contrasts with the strictly nonincreasing sequences established for the methods in [27,28]. The present nonmonotone condition on* $\left\{S_{t}\right\}$ *is also simpler to satisfy than its counterpart in those references. Moreover, the incorporation of a nonmonotone* $\left\{S_{t}\right\}$ *into the development of a new algorithm for solving (4) necessitates a different approach to establishing convergence.*

In [31], Nocedal observed that step sizes in nonmonotone line search methods can deviate unpredictably from unity, varying above or below one depending on problem scaling. Following this insight, and analogous to the strategy of employing larger step sizes for updating Lagrange multipliers in [32], we introduce a relaxation factor β>0 to update $W_{t+1}$ as


$$\begin{array}{c} W_{t+1}=Z_{t}+\beta \lambda_{t}D_{t}. \end{array}$$ (17)

The parameter β is empirically selected from the interval (1, 2).

The following lemma 2 is central to our global convergence analysis.

**Lemma 2** *For all* $\alpha \in (0,\alpha_{max}]$*,* W≥0*,*

(i) $\langle \nabla f(Z),D_{t}\rangle \leq -\frac{1}{\alpha }\|D_{t}{\|}^{2}\leq -\frac{1}{\alpha_{max}}\|D_{t}{\|}^{2}$,
(ii) *Z is a stationary point of (3) if and only if* $D_{t}=0,$
(iii) $\|D_{t}\|\leq \alpha_{t}\|\nabla f(Z_{t})\|.$

***Proof.*** The proof of (i) and (ii) are similar to the proof of Lemma 2.1 in [33] and therefore it is omitted here.

(iii) By use of the Cauchy equality, from (i), we obtain (iii). □

The following lemma is drown from Lemma 3 [15].

**Lemma 3** [15] *Assume that the sequences* $\left\{Z_{t}\right\}$ *and* $\left\{W_{t}\right\}$ *are generated by Algorithm 1, then we have*


$$\begin{array}{c} f(Z_{t})\leq f(W_{t})-\frac{L}{2}\|Z_{t}-W_{t}{\|}^{2}. \end{array}$$ (18)

To demonstrate that Algorithm 1 is well-defined, we establish the following result.

**Lemma 4** *For a given iterate* $Z_{t}$ *and a search direction* $D_{t}$ *at* $Z_{t}$*, there exists a step size* $\lambda_{t}$ *such that for all* t∈J*, the inequality*


$$\begin{array}{c} f(Z_{t}+\beta_{t}\lambda_{t}D_{t})\leq f(Z_{t})+\gamma \beta \lambda_{t}\langle \nabla f(Z_{t}),D_{t}\rangle \end{array}$$ (19)

*holds, where* $\lambda_{t}=\lambda \rho^{m}$.

***Proof.*** Assume no step size $\lambda_{t}$ satisfies inequality (19). Then for all sufficiently large positive integers *m*, we have


$$\begin{array}{c} f(Z_{t}+\beta \rho^{m}D_{t})-f(Z_{t})>\gamma \beta \rho^{m}\langle \nabla f(Z_{t}),D_{t}\rangle . \end{array}$$

By the mean value theorem, there exists $\theta_{t}\in (0,1)$ such that


$$\begin{array}{c} \beta \rho^{m}\langle \nabla f(Z_{t}+\theta_{t}\beta \rho^{m}D_{t}),D_{t}\rangle >\gamma \beta \rho^{m}\langle \nabla f(Z_{t}),D_{t}\rangle , \end{array}$$

which simplifies to


$$\begin{array}{c} \langle (\nabla f(Z_{t}+\theta_{t}\beta \rho^{m}D_{t})-\nabla f(Z_{t})),D_{t}\rangle >(\gamma -1)\langle \nabla f(Z_{t}),D_{t}\rangle . \end{array}$$

Taking the limit as m→∞ yields


$$\begin{array}{c} (\gamma -1)\langle \nabla f(Z_{t}),D_{t}\rangle \leq 0. \end{array}$$

Given γ∈(0,1), this implies $\langle \nabla f(Z_{t}),D_{t}\rangle \geq 0$, contradicting the condition $\langle \nabla f(Z_{t}),D_{t}\rangle \leq 0$. □

The following lemma implies that $S_{l(i)}$ is monotonically decreasing for all i∈{1,2,⋯,t} when i∈I.

**Lemma 5** *Suppose the sequence* $\left\{Z_{i}\right\}$ *for* i∈{1,2,⋯,t} *is generated by Algorithm 1; if* i∈I *holds for all* i∈{1,2,⋯,t}*, then* $S_{l(i)}\leq S_{l(i-1)}$*.*

***Proof.*** For i=1∈I, Lemma 3 implies that


$$\begin{array}{c} f(Z_{1})\leq f(W_{1})=f(Z_{0}). \end{array}$$ (20)

By the definition of $S_{t}$, we obtain


$$\begin{array}{c} \begin{array}{cc} S_{1} & =f(Z_{1})+\eta_{0}(S_{0}-f(Z_{1}))\\ & =(1-\eta_{0})f(Z_{1})+\eta_{0}S_{0}\\ & \leq (1-\eta_{0})f(Z_{0})+\eta_{0}S_{0}\\ & =S_{0}. \end{array} \end{array}$$ (21)

The inequality follows from (20). From (21) and the definition of $S_{l(t)}$, it follows that $S_{l(1)}\leq S_{l(0)}$, which establishes the result.

Next, we assume that for all *i* with 1≤i<t, the inequality $S_{i}\leq S_{i-1}$ holds; then, by the definition of $S_{l(t)}$, it follows that $S_{l(i)}\leq S_{l(i-1)}$. If $S_{i}\leq S_{i-1}$, the definition of $S_{i}$ yields $f(Z_{i})+\eta_{i-1}(S_{i-1}-f(Z_{i}))\leq S_{i-1}$, which implies


$$\begin{array}{c} f(Z_{i})\leq S_{i-1}. \end{array}$$ (22)

From the definition of $S_{i}$ together with (22), we obtain


$$\begin{array}{c} f(Z_{i})\leq S_{i}. \end{array}$$ (23)

For i=t∈I, the definition of $S_{t}$ and Lemma 3 give


$$\begin{array}{cc} S_{t}= & f(Z_{t})+\eta_{t-1}(S_{t-1}-f(Z_{t}))\\ & \leq (1-\eta_{t-1})f(Z_{t-1})+\eta_{t-1}S_{t-1}\\ & \leq (1-\eta_{t-1})S_{t-1}+\eta_{t-1}S_{t-1}\\ = & S_{t-1}. \end{array}$$

Here, the final equality relies on (23). By the definition of $S_{l(t)}$, we conclude that $S_{l(t)}\leq S_{l(t-1)}$. □

Next, we demonstrate a key property of the modified line search. For a given *W*_0_ > 0, the level set is defined as


$$\begin{array}{c} ℒ(W_{0})=\{W|f(W)\leq f(W_{0}),\;W\geq 0\}. \end{array}$$

**Lemma 6** *Suppose the sequences* $\left\{Z_{t}\right\}$ *and* $\left\{W_{t}\right\}$ *are generated by Algorithm 1; then* $\left\{Z_{t}\},\{W_{t}\}\subset ℒ(W_{0}\right)$*, and* $\left\{S_{l(t)}\right\}$ *is a decreasing sequence.*

***Proof.*** We first show that $\left\{S_{l(t)}\right\}$ is decreasing by considering two cases.

Case I: For t+1∈J with t∈{0,1,2,…}, the inequality m(t+1)≤m(t)+1 together with the definition of $\left\{S_{l(t)}\right\}$ and equation (34) implies


$$\begin{array}{c} \begin{array}{cc} S_{l(t+1)} & =\max_{0\leq j\leq m(t+1)}\{S_{t+1-j}\}\\ & \leq \max_{0\leq j\leq m(t)+1}\{S_{t+1-j}\}\\ & =\max\{S_{l(t)},S_{t+1}\}\\ & \leq S_{l(t)}. \end{array} \end{array}$$ (24)

Case II: For t+1∈I, the definition of $S_{t+1}$ gives


$$\begin{array}{c} \begin{array}{cc} S_{t+1} & =f(Z_{t+1})+\eta_{t}(S_{t}-f(Z_{t+1}))\\ & =(1-\eta_{t})f(Z_{t+1})+\eta_{t}S_{t}\\ & \leq (1-\eta_{t})f(Z_{t})+\eta_{t}S_{t}. \end{array} \end{array}$$ (25)

If t∈J, Algorithm 1 ensures $f(Z_{t})<S_{t-1}$, and since $S_{t-1}\leq S_{l(t)}$ by definition, we have


$$\begin{array}{c} f(Z_{t})\leq S_{l(t)}. \end{array}$$ (26)

Substituting inequality (26) into (25) yields


$$\begin{array}{c} \begin{array}{cc} S_{t+1} & \leq (1-\eta_{t})S_{l(t)}+\eta_{t}S_{t}\\ & \leq S_{l(t)}, \end{array} \end{array}$$ (27)

where the last step uses the definition of $S_{l(t)}$. Hence,


$$\begin{array}{c} S_{l(t+1)}\leq S_{l(t)}. \end{array}$$ (28)

If i∈I for i∈{t,t−1,…,t−j−1} and t−j∈J, then from Algorithm 1,


$$\begin{array}{c} f(Z_{t-j})<S_{t-j-1}. \end{array}$$ (29)

Combining this with (22) and (23) gives $S_{t-j+1}\leq S_{t-j}$, and as in the proof of Lemma 5, we obtain $S_{t}\leq S_{t-1}\leq \ldots \leq S_{t-j-1}$, so $S_{l(t+1)}\leq S_{l(t)}$ by definition. Otherwise, if i∈I for all i∈{t,t−1,…,0}, Lemma 5 again implies $S_{l(t+1)}\leq S_{l(t)}$.

The sequence $\left\{S_{l(t)}\right\}$ is therefore monotonically decreasing. We next demonstrate that $\left\{Z_{t}\right\}$ and $\left\{W_{t}\right\}$ lie within $ℒ(W_{0})$. Since $S_{l(0)}=f(Z_{0})\leq f(W_{0})$ holds by definition, we proceed by induction to show that $f(W_{t})\in ℒ(W_{0})$ for all t∈𝐍. Assuming $f(W_{i})\in ℒ(W_{0})$ for i=1,2,⋯,t, we verify that $W_{t+1}\in ℒ(W_{0})$. This result is established by considering the following two cases:

Case I: For t+1∈J, where t∈{0,1,2,⋯}, we apply (35) and Lemma 2 to obtain


$$\begin{array}{c} f(W_{t+1})\leq S_{l(t)}+\gamma \beta \lambda_{t}\langle \nabla f(Z_{t}),D_{t}\rangle \leq S_{l(t)}. \end{array}$$ (30)

Combining inequality (30) with Lemma 6 yields


$$\begin{array}{c} f(W_{t+1})\leq S_{l(t)}\leq f(Z_{0})\leq f(W_{0}). \end{array}$$ (31)

Therefore, the point $W_{t+1}$ lies within $ℒ(W_{0})$.

Case II: When t+1∈I, we use (26), (29), (22), (23) and Lemma 5 to derive


$$\begin{array}{c} f(Z_{t})\leq S_{l(t)}. \end{array}$$ (32)

Algorithm 1 gives $f(W_{t+1})=f(Z_{t}).$ This equality, combined with (32) and Lemma 6, leads to


$$\begin{array}{c} f(W_{t+1})\leq S_{l(t)}\leq f(Z_{0})\leq f(W_{0}). \end{array}$$ (33)

Consequently, the point $W_{t+1}$ also belongs to $ℒ(W_{0})$. Thus, the entire sequence $\left\{W_{t}\right\}$ remains within $ℒ(W_{0})$. Lemma 3 further confirms that $\left\{Z_{t}\}\subset ℒ(W_{0}\right)$. □

The following corollary establishes the convergence of the sequence $\left\{S_{l(t)}\right\}$.

**Corollary 1** *Suppose that the level set* $ℒ(W_{0})$ *is bounded; then the sequence* $\left\{S_{l(t)}\right\}$ *converges.*

***Proof***. From (31), (33), and Lemma 6, it follows that there exists τ such that for all n∈𝐍,


$$\begin{array}{c} \tau \leq f(W_{t+n})\leq S_{l(t+n)}\leq S_{l(t-1+n)}\leq \cdots \leq S_{l(t+1)}\leq S_{l(t)}, \end{array}$$

which implies that the sequence $\left\{S_{l(t)}\right\}$ is bounded below. Therefore, $\left\{S_{l(t)}\right\}$ converges. □

We now establish the well-definiteness of the line search.

**Theorem 1** *Suppose the sequences* $\left\{Z_{t}\right\}$ *and* $\left\{W_{t}\right\}$ *are generated by Algorithm 1. Then Algorithm 1 is well-defined.*

***Proof***. The proof is straightforward for all t∈I, so it suffices to show that


$$\begin{array}{c} S_{t+1}\leq S_{l(t)}+(1-\eta_{t})\gamma \beta {\tilde{\lambda }}_{t}\langle \nabla f(Z_{t}),D_{t}\rangle \end{array}$$ (34)

and


$$\begin{array}{c} f(Z_{t}+\beta \lambda_{t}D_{t})\leq S_{l(t)}+\gamma \beta \lambda_{t}\langle \nabla f(Z_{t}),D_{t}\rangle \end{array}$$ (35)

are well-defined for all t∈J. We proceed by induction. The base case *t* = 0 is easily verified. Now assume that for some $\lambda_{i}$ and all *i* with 0<i≤t, inequali*t*ies (34) and (35) hold.

From Algorithm 1, we have $f(Z_{t})<S_{t-1}$. Given that $S_{t-1}\leq S_{l(t)}$, it follows that $f(Z_{t})\leq S_{l(t)}$. For *i* = *t* + 1, Lemma 4 guarantees the existence of a step size ${\tilde{\lambda }}_{t}>0$ such that (19) holds. According to Lemma 3, we derive


$$\begin{array}{cc} \left(1-\eta_{t})f(Z_{t+1}\right) & \leq (1-\eta_{t})f(Z_{t})+(1-\eta_{t})\gamma \beta {\tilde{\lambda }}_{t}\langle \nabla f(Z_{t}),D_{t}\rangle \\ & \leq (1-\eta_{t})S_{l(t)}+(1-\eta_{t})\gamma \beta {\tilde{\lambda }}_{t}\langle \nabla f(Z_{t}),D_{t}\rangle \\ & \leq \eta_{t}(S_{l(t)}-S_{t})+(1-\eta_{t})S_{l(t)}+(1-\eta_{t})\gamma \beta {\tilde{\lambda }}_{t}\langle \nabla f(Z_{t}),D_{t}\rangle . \end{array}$$

The final inequality is equivalent to (34), confirming that (34) is well defined. From (19), we observe that $\lambda_{t}$ satisfies


$$\begin{array}{ccc} f(Z_{t}+\beta \lambda_{t}D_{t}) & \leq & f(Z_{t})+\gamma \beta \lambda_{t}\langle \nabla f(Z_{t}),D_{t}\rangle \\ & \leq & S_{l(t)}+\gamma \beta \lambda_{t}\langle \nabla f(Z_{t}),D_{t}\rangle . \end{array}$$

This last inequality ensures that (35) is well defined. Consequently, Algorithm 1 is well-defined. □

We now introduce a key property of the sequence $S_{l(tM)}$, which is essential for establishing global convergence.

**Theorem 2** *Assume that the sequences* $\left\{Z_{t}\right\}$ *and* $\left\{W_{t}\right\}$ *are generated by Algorithm 1; then,*


$$\begin{array}{c} S_{l((t+1)M)}\leq S_{l(tM)}+\delta_{l((t+1)M)-1}\lambda_{l((t+1)M)-1}\langle \nabla f(Z_{l((t+1)M)-1}),D_{l((t+1)M)-1}\rangle , \end{array}$$ (36)

*where* $\delta_{l((t+1)M)-1}=\gamma \beta (1-\eta_{l((t+1)M)-1})$ *and* t∈{1,2,⋯}*. Furthermore,*


$$\begin{array}{c} \sum_{t=1}^{\infty }\lambda_{l((t+1)M)-1}|\langle \nabla f(Z_{l((t+1)M)-1}),D_{l((t+1)M)-1}\rangle |<\infty . \end{array}$$ (37)

***proof*** From the definition of $S_{l(t)}$ and Lemma 6, we obtain


$$\begin{array}{cc} S_{tM+1} & \leq \max\{S_{(t-1)M+1},\cdots ,S_{tM}\}+\delta_{tM}\lambda_{tM}\langle \nabla f(Z_{tM}),D_{tM}\rangle \\ & =S_{l(tM)}+\delta_{tM}\lambda_{tM}\langle \nabla f(Z_{tM}),D_{tM}\rangle \\ S_{tM+2} & \leq \max\{S_{l(tM)},S_{tM+1}\}+\delta_{tM+1}\lambda_{tM+1}\langle \nabla f(Z_{tM+1}),D_{tM+1}\rangle \\ & =S_{l(tM)}+\delta_{tM+1}\lambda_{tM+1}\langle \nabla f(Z_{tM+1}),D_{tM+1}\rangle \\ \vdots \end{array}$$

By induction,


$$\begin{array}{c} S_{tM+i}\leq S_{l(tM)}+\delta_{tM+i-1}\lambda_{tM+i-1}\langle \nabla f(Z_{tM+i-1}),D_{tM+i-1}\rangle \end{array}$$

for i∈{1,2,⋯,M}. Because l((t+1)M)∈{tM+1,⋯,(t+1)M}, the desired result (36) follows. We now prove (37). Since *f* is bounded below and $S_{l(tM)}$ is a convex combination of certain values of $f(Z_{tM})$, the sequence $S_{l(tM)}$ is also bounded below. Combining the inequalities in (36) with Corollary 1 yields


$$\begin{array}{cc} \sum_{t=1}^{\infty }\delta_{l(tM)-1}|\langle \nabla f(Z_{l(tM)-1}),D_{l(tM)-1}\rangle | & \leq S_{l(0)}-\lim_{t\to \infty }S_{l(tM)}\\ & <\infty . \end{array}$$

This completes the proof.

### 2.2 Convergence analysis

This section analyzes the convergence properties of the proposed algorithm, establishing its global convergence. To prove the main result, we first derive a lower bound for the step length $\lambda_{t}$.

**Lemma 7** *Assume* $\lambda_{t}$ *is a step size generated by Algorithm 1; if* $Z_{t}$ *is not a stationary point of (3), then for all* t∈J*, there exists a constant* $\tilde{\lambda }$ *such that* $\lambda_{t}\geq \tilde{\lambda }$*.*


$$\begin{array}{c} \lambda_{t}\geq \min\{1,\frac{2\rho (1-\gamma )}{L\alpha_{max}}\}. \end{array}$$ (38)

***Proof.*** For the obtained step size $\lambda_{t}$, if $\lambda_{t}$ does not satisfy (35), that is,


$$\begin{array}{ccc} f(Z_{t}+\beta_{t}\lambda_{t}D_{t}) & \geq & S_{l(t)}+\gamma \beta \lambda_{t}\langle \nabla f(Z_{t}),D_{t}\rangle \\ & \geq & f(Z_{t})+\gamma \beta \lambda_{t}\langle \nabla f(Z_{t}),D_{t}\rangle . \end{array}$$

Thus,


$$\begin{array}{c} f(Z_{t}+\beta_{t}\lambda_{t}D_{t})\geq f(Z_{t})+\gamma \beta \lambda_{t}\langle \nabla f(Z_{t}),D_{t}\rangle . \end{array}$$

Similar to Lemma 4 in [15], we obtain


$$\begin{array}{c} \lambda_{t}\geq \min\{1,\frac{2\rho (1-\gamma \beta )}{L\alpha_{max}}\}:=\tilde{\lambda }. \end{array}$$

Inequality (38) is therefore satisfied. To establish global convergence, we now prove the following result.

**Lemma 8** *Assume that the sequences* $\left\{Z_{t}\right\}$ *and* $\left\{W_{t}\right\}$ *are generated by Algorithm 1. Then*


$$\begin{array}{c} \|\nabla f(W_{t+1})\|\leq (1+\alpha_{max}\lambda_{max}\beta L)\|\nabla f(Z_{t})\|. \end{array}$$ (39)

*Moreover,*


$$\begin{array}{c} \lim_{t\to \infty }\|D_{l(tM)-1}\|=0. \end{array}$$ (40)

***Proof.*** Algorithm 1 ensures that the step size satisfies $\lambda_{t}\leq \lambda_{max}$ for all t∈J. Using Lemma 2(iii), we obtain


$$\begin{array}{c} \|W_{t+1}-Z_{t}\|=\beta \lambda_{t}\|D_{t}\|\leq \alpha_{max}\lambda_{max}\beta \|\nabla f(Z_{t})\|. \end{array}$$ (41)

The Lipschitz continuity of ∇f implies


$$\begin{array}{c} \left\|\nabla f(W_{t+1})-\nabla f(Z_{t})\|\leq \alpha_{max}\lambda_{max}\beta L\|\nabla f(Z_{t})\right\| \end{array}$$ (42)

Therefore, inequality (39) is established.

We now demonstrate the validity of equality (40).

Using Theorem 2, it follows that


$$\begin{array}{cc} \sum_{t=2}^{\infty }\tilde{\lambda }\frac{1}{\alpha_{max}}\|D_{l(tM)-1}{\|}^{2} & \leq \sum_{t=2}^{\infty }\tilde{\lambda }|\langle \nabla f(Z_{l(tM)-1}),D_{l(tM)-1}\rangle |\\ & \leq \sum_{t=2}^{\infty }\lambda_{l(tM)-1}|\langle \nabla f(Z_{l(tM)-1}),D_{l(tM)-1}\rangle |\\ & <\infty . \end{array}$$

Thus


$$\begin{array}{c} \lim_{t\to \infty }\|D_{l(tM)-1}\|=0. \end{array}$$

We next establish the following global convergence result for our HMNMF method. □

**Theorem 3** *Suppose that the level set* $ℒ(W_{0})$ *is bounded; then any accumulation point of the sequence* $\left\{Z_{t}\right\}$ *generated by Algorithm 1 is a stationary point of (3).*

***Proof.*** We consider two cases.

**Case I:** For t∈I, Lemma 8 implies that


$$\begin{array}{c} \lim_{t\to \infty }\|D_{t}\|=0, \end{array}$$ (43)

so any accumulation point of $\left\{Z_{t}\right\}$ is a stationary point of (3).

**Case II:** For t∈J, the boundedness of $ℒ(W_{0})$ together with Lemma 8 and Lemma 2(ii) shows that $\left\{Z_{l(tM)-1}\right\}$ has an accumulation point $Z_{*}$ that is a stationary point of (3). We now prove Theorem 3 by contradiction. Assume $Z_{*}$ is not a stationary point of (3). Then Lemma 2(ii) gives $\|D_{\alpha }(Z_{*})\|>0$ for all $\alpha \in (0,\alpha_{max}]$. By continuity and compactness, there exists η>0 such that $\|D_{\alpha }(Z_{*})\|>\eta$ for $\alpha \in [\alpha_{min},\alpha_{max}]$. Hence, for sufficiently large *t*, $\|D_{\alpha }(Z_{*})\|>\eta /2$. Using Lemmas 2, 3, 6 and 7, we derive


$$\begin{array}{cc} f(Z_{l(tM)}) & \leq f(W_{l(tM)})\\ & =f(Z_{l(tM)-1}+\beta \lambda_{l(tM)-1}D_{l(tM)-1})\\ & \leq S_{l(tM)-1}+\gamma \beta \lambda_{l(tM)-1}\langle \nabla f(Z_{l(tM)-1}),D_{l(tM)-1}\rangle \\ & \leq S_{l(tM)-1}-\frac{\gamma \beta \tilde{\lambda }}{\alpha_{max}}\|D_{l(tM)-1}{\|}^{2}\\ & \leq S_{l(0)}-\frac{\gamma \beta \tilde{\lambda }\eta^{2}}{4\alpha_{max}} \end{array}$$

which yields


$$\begin{array}{c} f(Z_{l(tM)})\to -\infty \;\text{as}\;t\to \infty . \end{array}$$

This contradicts the property of the norm, so $Z_{*}$ must be a stationary point of (3).

By Theorem 3, Lemma 2, and Lemma 3, the following key convergence result is established.

**Theorem 4** *If the level set* $ℒ(W_{0})$ *is bounded, then every accumulation point of the sequence* $\left\{W_{t}\right\}$ *produced by Algorithm 1 constitutes a stationary point of (3).*

## 3 Numerical experiments

This section presents numerical experiments evaluating our algorithm’s performance against six methods: NeNMF [14], the projected BB method (APBB2 [13]) (The code is available at http://homepages.umflint.edu/~lxhan/software.html), QRPBB [15], MPBB [16], hierarchical alternating least squares (HALS) [34], and the block coordinate descent (BCD) method [30]. We compared these methods using both synthetic datasets and real-world problems from the Yale image database (http://www.cad.zju.edu.cn/home/dengcai/Data/TextData.html), the CBCL image database http://cbcl.mit.edu/software-datasets/FaceData2.html, and the Reuters-21578 and TDT2 text corpora (Both Reuters-21578 corpus and TDT-2 corpus in MATLAB format are available at http://www.cad.zju.edu.cn/home/dengcai/Data/TextData.html). All code was implemented in MATLAB. The experiments ran on a Lenovo laptop with an Intel Core(TM) i7 CPU at 4.9 GHz and 16 GB of memory, using Windows 10 and MATLAB R2021a.

### 3.1 Stopping criterion

By the KKT conditions for constrained optimization, (*W*,*H*) is stationary point of NMF (2) if and only if it satisfies $\nabla_{W}^{P}f(W,H)=0$ and $\nabla_{H}^{P}f(W,H)=0$, where


$$[\nabla_{W}^{P}f(W,H){]}_{ij}=\left\{[\nabla_{W}f(W,H){]}_{ij},\;\;\;\;\;\;\;\;\;\;\;\;\;\;\;\mathrm{if}\;\;W_{ij}>0,\;\;\;\min\{0,[\nabla_{W}f(W,H){]}_{ij}\},\;\;\;\mathrm{if}\;\;W_{ij}=0,\;\;\right)$$

and $\nabla_{H}^{P}f(W^{\left(k\right)},H^{\left(k\right)})$ is defined in the same way. Thus, we use the following stopping criterion as Lin [12] in the numerical experimentation:


$$\begin{array}{c} \begin{array}{cc} & \left\|[\nabla_{W}^{P}f(W^{\left(k\right)},H^{\left(k\right)}),\nabla_{H}^{P}f(W^{\left(k\right)},H^{\left(k\right)}{)}^{T}]\right\|\\ & \leq \epsilon \|[\nabla_{W}^{P}f(W^{\left(1\right)},H^{\left(1\right)}),\nabla_{H}^{P}f(W^{\left(1\right)},H^{\left(1\right)}{)}^{T}]\|, \end{array} \end{array}$$ (44)

where ϵ>0 is a tolerance.

### 3.2 Synthetic data

We first evaluate the performance of HMNMF and the other algorithms on synthetic datasets. The matrix *V* in this test is exactly low-rank and is constructed as *V* = *LR*, where *L* and *R* are generated using the MATLAB commands *max*(0,*randn*(*m*,*r*)) and *max*(0,*randn*(*r*,*n*)), respectively.

The parameters of our method are set as follows throughout all experiments: $\alpha_{\text{max}}=10^{20}$, $\alpha_{\text{min}}=10^{-20}$, ρ=0.25, $\gamma =10^{-4}$, and λ=1. These settings are consistent with those used in APBB2, QRPBB, and MPBB. The tolerance for all algorithms is set to tol = 10^−8^. For HMNMF, we choose β=1.88 and update $\eta_{t}$ according to equation (12). The maximum number of iterations (maxit) is set to 50,000 for all algorithms. All other parameters for APBB2, NeNMF, QRPBB, and MPBB are set to their default values.

For each test dataset with independent Gaussian noise at a signal-to-noise ratio of 60 dB, we use 10 different randomly generated initial points. The average results are summarized in Table 1. Here, “iter” denotes the number of iterations required to satisfy the termination criterion (44), and “niter” represents the total number of sub-iterations for solving subproblems (3) and (4). Performance is measured using the relative error $\text{relerr}=\|V-W_{k}H_{k}{\|}_{F}/\|V{\|}_{F}$, the final projected gradient norm $\|[\nabla_{P}^{H}F(W_{k},H_{k}),\nabla_{P}^{W}F(W_{k},H_{k})]{\|}_{F}$, and the CPU time (in seconds). As shown in Table 1, HMNMF achieves the shortest execution time and the smallest number of sub-iterations among all algorithms, particularly for large-scale datasets. These results indicate that HMNMF significantly outperforms the other four solvers across all test cases. This improvement can be attributed to two factors: first, the modified objective function in the nonmonotone line search $S_{l}(t)$ may enhance efficiency; second, the introduction of the relaxation factor β in the update rules allows larger step sizes, thereby reducing computational time.

**Table 1:** Experimental results on synthetic datasets.

(m n r)	Alg	Iter	Niter	Pgn	Time	Residual
(600 200 30)	NeNMF	153.3	5456.0	7.58E-4	1.52	7.5550E-4
	APBB2	161.8	3441.8	7.94E-4	2.13	7.5460E-4
	QRPBB	141.0	1988.4	5.60E-4	1.21	7.5509E-4
	MPBB	83.1	2547.9	6.86E-4	0.87	7.5306E-4
	HMNMF	52.2	530.4	6.84E-4	0.39	7.5449E-4
(500 700 40)	NeNMF	160.3	5807.8	2.46E-3	3.05	7.7824E-4
	APBB2	185.0	4545.3	2.18E-3	5.58	7.7758E-4
	QRPBB	147.9	2190.9	1.70E-3	2.69	7.7762E-4
	MPBB	97.9	3050.4	2.32E-3	2.09	7.7752E-4
	HMNMF	72.1	712.1	2.01E-3	1.03	7.7781E-4
(1000 200 50)	NeNMF	201.1	7784.6	2.58E-3	6.16	7.1992E-4
	APBB2	249.1	6443.7	2.57E-3	11.44	7.2020E-4
	QRPBB	240.4	3579.7	2.29E-3	5.00	7.1959E-4
	MPBB	133.7	4827.2	2.43E-3	4.04	7.1962E-4
	HMNMF	96.3	1010.6	2.28E-3	1.59	7.1969E-4
(1000 600 60)	NeNMF	226.2	8357.3	6.44E-3	8.82	7.7041E-4
	APBB2	241.5	6121.9	6.21E-3	15.73	7.7086E-4
	QRPBB	207.7	3375.3	5.63E-3	8.00	7.7116E-4
	MPBB	124.8	4386.5	6.39E-3	6.12	7.7124E-4
	HMNMF	88.8	974.7	5.16E-3	2.68	7.7098E-4
(500 2000 70)	NeNMF	240.8	10004.9	1.29E-2	30.25	7.6550E-4
	APBB2	251.1	6929.6	1.32E-2	37.32	7.6553E-4
	QRPBB	211.2	3492.2	1.10E-2	17.84	7.6549E-4
	MPBB	138.6	4611.8	1.25E-2	15.09	7.6589E-4
	HMNMF	97.6	1150.2	1.17E-2	6.85	7.6546E-4
(3000 900 80)	NeNMF	238.5	8946.3	3.13E-2	54.35	7.8611E-4
	APBB2	267.4	6860.5	3.24E-2	95.20	7.8594E-4
	QRPBB	262.6	4127.7	2.82E-2	48.05	7.8631E-4
	MPBB	165.0	5905.1	3.06E-2	40.17	7.8566E-4
	HMNMF	87.4	1059.4	2.55E-2	13.50	7.8602E-4
(5000 1000 90)	NeNMF	267.6	9911.9	6.03E-2	100.90	7.8828E-4
	APBB2	271.8	9894.6	5.55E-2	221.37	7.8828E-4
	QRPBB	223.2	4263.6	5.76E-2	91.66	7.8828E-4
	MPBB	162.5	5862.3	5.97E-2	80.61	7.8844E-4
	HMNMF	117.9	1455.1	5.79E-2	34.77	7.8828E-4
(8000 1000 100)	NeNMF	287.3	10579.7	1.47E-1	209.28	7.8661E-4
	APBB2	291.3	10939.6	1.14E-1	189.83	7.8661E-4
	QRPBB	223.2	4309.1	1.08E-1	154.14	7.8661E-4
	MPBB	165.5	5628.6	1.15E-1	139.37	7.8718E-4
	HMNMF	133.8	1715.6	1.11E-1	69.03	7.8661E-4

**Fig 1:**
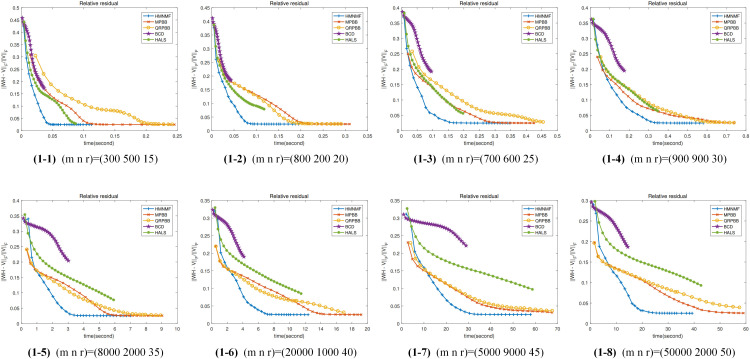
The influence of different choices of parameter $\eta_{t}$ on the computational efficiency of HMNMF on the synthetic datasets.

**Fig 2:**
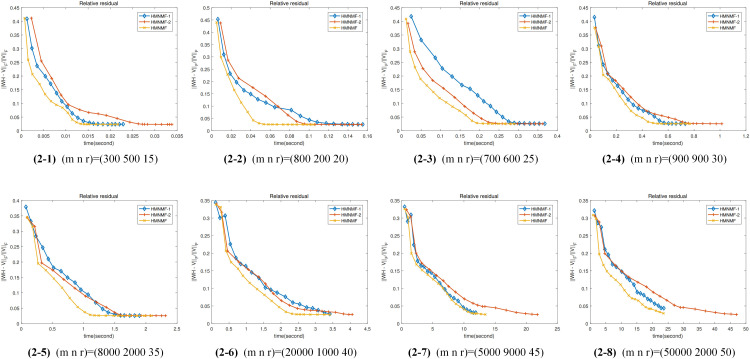
The influence of different relaxation factors S on the computational efficiency of HMNMF on the synthetic datasets.

**Fig 3:**
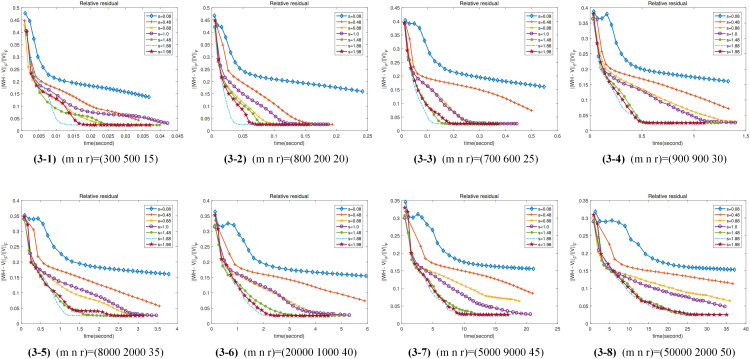
The influence of different relaxation factors S on the computational efficiency of HMNMF on the synthetic datasets.

With the close association between HMNMF, QRPBB, and MPBB, and considering HALS and BCD as some of the most efficient NMF methods, we further compare the performance of HMNMF, QRPBB, MPBB, HALS, and BCD. Figs 1–3 present comparisons of these five methods on eight randomly generated datasets with independent Gaussian noise at a signal-to-noise ratio of 30 dB. The algorithms are terminated when the stopping criterion in (44) is satisfied with $\epsilon =10^{-8}$ or when the iteration count exceeds 30. The results clearly demonstrate that HMNMF requires the fewest iterations and the least CPU time to reach the specified tolerance in most cases, especially for high-dimensional datasets. Moreover, for high-dimensional data, the PBB-based methods outperform HALS and BCD, which can be explained by the fact that PBB-based methods implicitly utilize second-order information of the cost function, whereas HALS and BCD rely on first-order approximations to search for stationary points.

### 3.3 Image data

The Yale face database, created by the Yale Center for Computational Vision and Control, contains 165 grayscale images of 15 individuals, with 11 images per person. The images exhibit variations in lighting conditions (left-light, center-light, right-light), facial expressions (normal, happy, sad, surprised, wink), and the presence or absence of glasses. The data matrix *V*, with rows representing images, has dimensions 165 × 4096.

The CBCL image database comprises 2,429 facial images, each with 19×19 pixels, resulting in a matrix of size 361×2,429.

For each database, we perform 10 trials with different randomly generated initial points, using $\epsilon =10^{-8}$ in (44) and a maximum of 50,000 iterations for all algorithms. The average results are presented in Table 2. It can be observed that for both databases, HMNMF converges in fewer iterations and less CPU time, while achieving residual values comparable to those of other solvers.

**Table 2:** Experimental results on Yale and CBCL datasets.

(m n r)	Alg	Iter	Niter	Pgn	Time	Residual
(165 4,096 20)	NeNMF	3122.2	127172.0	1.12E-1	122.34	1.849E-1
	APBB2	2896.8	93407.0	1.04E-1	209.27	1.848E-1
	QRPBB	2632.5	51428.7	9.38E-2	88.92	1.848E-1
	MPBB	1725.0	66687.0	1.02E-1	65.18	1.846E-1
	HMNMF	812.7	9104.7	1.09E-1	16.78	1.848E-1
(361 2,429 49)	NeNMF	1767.3	99033.1	2.09E-2	153.26	1.925E-1
	APBB2	395.7	18474.5	1.87E-2	29.18	1.926E-1
	QRPBB	540.8	12244.8	1.68E-2	20.66	1.929E-1
	MPBB	593.5	20806.6	1.78E-2	32.97	1.926E-1
	HMNMF	284.7	5707.3	1.60E-2	14.59	1.924E-1

### 3.4 Text data

The Reuters-21578 corpus includes 21,578 documents distributed across 135 categories. Documents with multiple category labels were discarded, leaving 8,293 documents in 65 categories. After preprocessing, the corpus contains 18,933 distinct terms, represented by a matrix of size 18,933×8,293.

The TDT2 corpus consists of news articles from sources such as ABC, CNN, VOA, NYT, PRI, and APW from 1998. It contains 11,201 on-topic documents classified into 96 semantic categories. After removing documents appearing in two or more categories and retaining only the largest 30 categories, we obtain 9,394 documents, represented by a matrix of size 36,771×9,394.

As in the previous subsection, we set $\epsilon =10^{-8}$, and report average results over 10 random initializations in Table 3. The results show that the CPU time required by HMNMF is significantly less than that of NeNMF, APBB2, QRPBB, and MPBB. Meanwhile, the projected gradient norms obtained by APBB2, QRPBB, MPBB, and HMNMF are similar and smaller than those of NeNMF, which confirms the effectiveness of our proposed HMNMF method.

**Table 3:** Experimental results on Reuters-21578 and TDT2 datasets.

(m n r)	Alg	Iter	Niter	Pgn	Time	Residual
(18,933 8,293 65)	NeNMF	397.5	18660.0	1.34E + 0	476.06	6.877E-1
	APBB2	161.3	3691.9	1.01E + 0	265.84	6.885E-1
	QRPBB	123.6	2015.3	1.06E + 0	116.98	6.889E-1
	MPBB	152.0	4926.1	1.13E + 0	154.60	6.879E-1
	HMNMF	46.0	478.7	1.03E + 0	28.83	6.878E-1
(36,771 9,394 100)	NeNMF	206.7	7936.1	4.88E + 0	560.75	7.396E-1
	APBB2	108.1	1967.6	3.78E + 0	380.35	7.398E-1
	QRPBB	120.4	1328.5	3.01E + 0	233.38	7.396E-1
	MPBB	54.3	1376.7	3.67E + 0	115.43	7.399E-1
	HMNMF	40.1	303.8	4.00E + 0	56.35	7.398E-1

### 3.5 Influence of parameters $\eta_{t}$ and β

**Fig 4:**
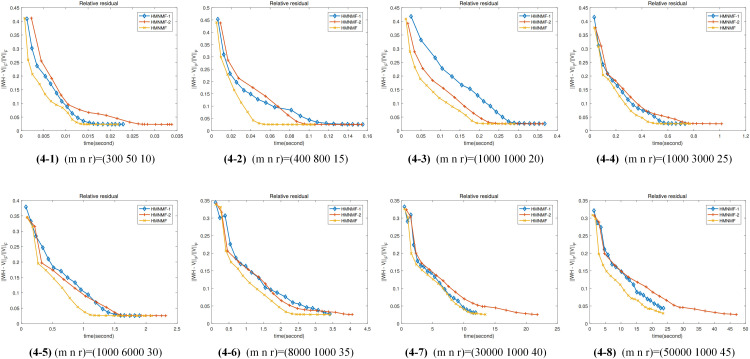
Residual value versus time of HMNMF on synthetic datasets.

In this section, we first intend to investigate what is the impact for our modified nonmonotone line search rules. In particular, how does the definition of $\eta_{t}$ in (10) improve the numerical efficiency of algorithms? To do so, we shall compare our $\eta_{t}$ with the other two $\eta_{t}$, proposed in [28,29]. For simplicity of statement, we introduce the following notations. HMNMF-1:Algorithm 1 with the $\eta_{t}$ defined in [28]; HMNMF-2:Algorithm 1 with the $\eta_{t}$ defined in [29]; HMNMF:Algorithm 1 with the $\eta_{t}$ defined by (12). Fig 4 shows the relative residual value versus the running time on synthetic datas which are the same as those in Section 3.1. The maximum number of iterations (maxit) is set to be 20 for all algorithms, and all of the other parameters for experimental settings here are the same as those in Section 3.1. From Fig 3, it is clear that our $\eta_{t}$ is superior to the other two $\eta_{t}$ in [28,29].

**Fig 5:**
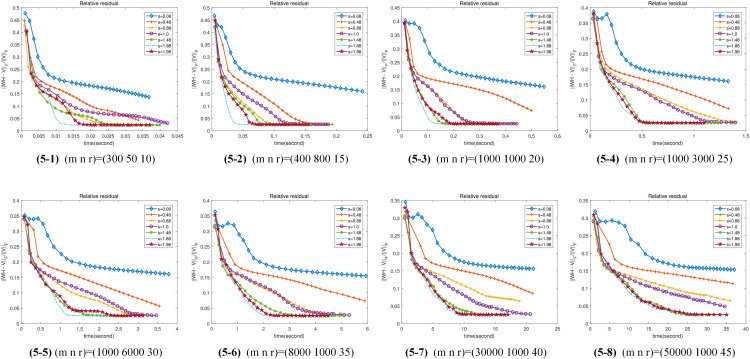
Residual value versus time of HMNMF on relaxation factor s on synthetic datasets.

Next we report that how does the adjustability of the relaxation factor *s* in the update rule of $W_{t+1}$ can improve the numerical efficiency of algorithms? In fact, we run the HMNMF with the following different *s*: *s* = 0.08, 0.48, 0.88, 1.0, 1.48, 1.88, 1.98 on synthetic datas which are the same as those in Section 3.1. Fig 5 shows the relative residual value versus the running time. The maximum number of iterations (maxit) is set to be 30 for all algorithms, and all of the other parameters for experimental settings here are the same as those in Section 3.1. From Fig 5, we can see that the relaxation factor *s* fails to accelerate the convergence when *s* < 1 and increasing constant *s* significantly accelerates the convergence when 1 < *s* < 2. As for NMPBB, it is seem that *s* = 1.88 is much better value than the other values in term of convergence rate, thus we use *s* = 1.88 for our HMNMF in all experiments.

## 4 Conclusion

This paper introduces a fast prox-linear variant of a hybrid algorithm for solving NMF within the ANLS framework; the algorithm employs either a modified nonmonotone projected Barzilai-Borwein gradient method or a block coordinate descent method to address the subproblems in each iteration. A modified line search rule incorporating a nonmonotone technique [25,27] is also integrated into the algorithm to enhance its efficiency. Under mild assumptions, the global convergence of the algorithm is established. Numerical experiments demonstrate the effectiveness of the proposed algorithm for NMF.
